# Next-generation Mobile Cardiac Telemetry: Clinical Value of Combining Electrocardiographic and Physiologic Parameters

**DOI:** 10.19102/icrm.2022.130807

**Published:** 2022-08-15

**Authors:** Dale Yoo, Karan Bhalla, Harish Manyam, Dinesh Pubbi, Ira H. Lieber

**Affiliations:** ^1^Heart Rhythm Specialists, PLLC, Dallas, TX, USA; ^2^ORION MEDICAL, Comprehensive Cardiovascular, Sleep Medicine and IAC Accredited Vein Center, Pasadena, TX, USA; ^3^Erlanger Heart and Lung Institute, University of Tennessee, Chattanooga, TN, USA; ^4^First Coast Heart and Vascular Center, St. Augustine, FL, USA; ^5^Texas Cardiology Associates of Houston, Kingwood, TX, USA

**Keywords:** Biometrics, patient management, remote monitoring

## Abstract

The ZOLL Arrhythmia Management System, a mobile cardiac telemetry (MCT) device from ZOLL Corporation (Chelmsford, MA, USA), records single-channel electrocardiogram (ECG) signals, heart rate, activity, respiratory rate, and posture. Comprehensive reporting from these multiple biometrics may provide a global evaluation of arrhythmic or other cardiovascular risks in individual patients and insights into the patient’s overall wellness and health status. The objective of the study was to evaluate the physician-perceived utility of adding biometric data to the traditional ECG-only–based assessment and subject-reported symptoms. This prospective study recruited candidates for MCT. Independent event and end-of-use (EOU) reports based on ECG and biometrics data were provided to physicians. To document whether the biometric data affected treatment plan decisions or added value over the ECG-alone data, physicians completed a questionnaire for each report. Additionally, they completed the questionnaire to understand the utility of the subject wellness information provided in the EOU report. From December 2020 to July 2021, 583 patients were enrolled by 27 physicians from 18 cardiology practices in the United States. When using biometrics data compared to the ECG alone, this study found that 96% of the physicians made changes to the treatment plan that initially was based on the ECG alone. The biometrics-based changes involved 64% of all patients (n = 535), and included modifications to medications, follow-up, and lifestyle in 18%, 19%, and 63% of the subjects, respectively. In this largest MCT study conducted to date, next-generation MCT, by providing multiple biometric parameters along with ECG data, improves physicians’ ability to make patient management decisions. This added functionality and clarity may replace traditional “ECG with diary”–based monitoring.

## Introduction

While the ability to record arrhythmic events has improved in both clarity and duration with the current remote arrhythmia-monitoring systems, there is an unmet clinical need for understanding the provocative factors and subsequent consequences associated with arrhythmias and symptoms. Currently, understanding these factors relies on diaries, which are subjective and often either not completed, lost, or of insufficient accuracy to be useful.^1,2^ For example, without a highly accurate patient diary, it is currently not possible to know if a person’s episodes of atrial fibrillation (AF) occur only during exercise, or, alternatively, whether someone’s sinus pauses only occur during sleep, which could be daytime sleep. The ability to provide objective physiologic data could reduce or eliminate the reliance on diary information for rhythm and symptom interpretation. Additionally, previous studies have shown that 12%–50% of patients experience no arrhythmias or symptoms.^3^ In such cases, it is also hypothesized that, during the typical 30-day duration for mobile cardiac telemetry (MCT), longitudinal tracking of physiologic data may help clinicians. This may include the discovery of a disordered sleep pattern in a patient complaining of fatigue, or significant decreases in activity during bouts of paroxysmal AF or heart failure.

The ZOLL Arrhythmia Management System (AMS) (ZOLL Corporation, Chelmsford, MA, USA) is a wearable patch with standard MCT indications cleared by the U.S. Food and Drug Administration. While the AMS device has all the capabilities of a standard electrocardiogram (ECG)-based MCT, it also has the ability to report heart rate (HR), activity, respiratory rate (RR), and body posture. A comprehensive report incorporating ECGs, symptoms, and biometrics data is provided to medical professionals. The report not only allows clinicians to diagnose and treat rhythm disorders but also provides contextual information around the reported events (both arrhythmias and patient-triggered symptoms). Thus, it is anticipated that such a comprehensive analysis from multiple biometrics parameters will provide a global evaluation of arrhythmic or other cardiovascular risks in individual patients and insights into the patient’s overall wellness and health status.

To determine the aforementioned potential benefits of the AMS, this study was designed for several purposes: first, to determine associations among biometric data with arrhythmias and subject-reported symptoms; second, to evaluate the clinical utility, as determined by the prescribing physicians, provided by adding biometric information to the ECG-only and/or symptom-only event assessment; and third, to evaluate the usefulness of longitudinal information over the course of the monitoring duration to assist in patient management decisions.

## Methods

**Figure 1:**
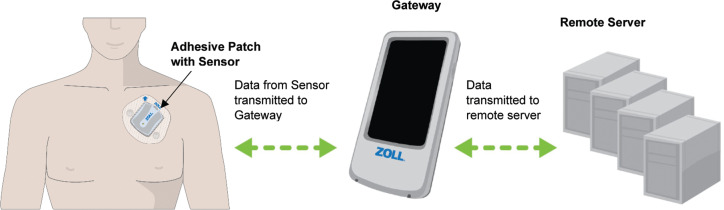
The ZOLL Arrhythmia Management System consists of an adhesive patch, sensor, gateway, and remote server.

The AMS sensor along with the adhesive patch is self-applied to provide a lead II–equivalent electrode placement **(Figure 1)**. Replacement of the patch and recharging (approximately 45 min) of the sensor are typically done every 5 days. The sensor automatically acquires ECG, HR, RR, activity, and posture measurements. When patients experience a symptom, they can use the provided dedicated cellular gateway to record the event and select the symptom that best describes their current state. All data are transmitted at regular intervals from the sensor to the gateway, then from there to the server for analysis. After review by certified cardiac technicians, the clinical values are used for event reports, daily reports, and end-of-use (EOU) reports.

**Figure 2:**
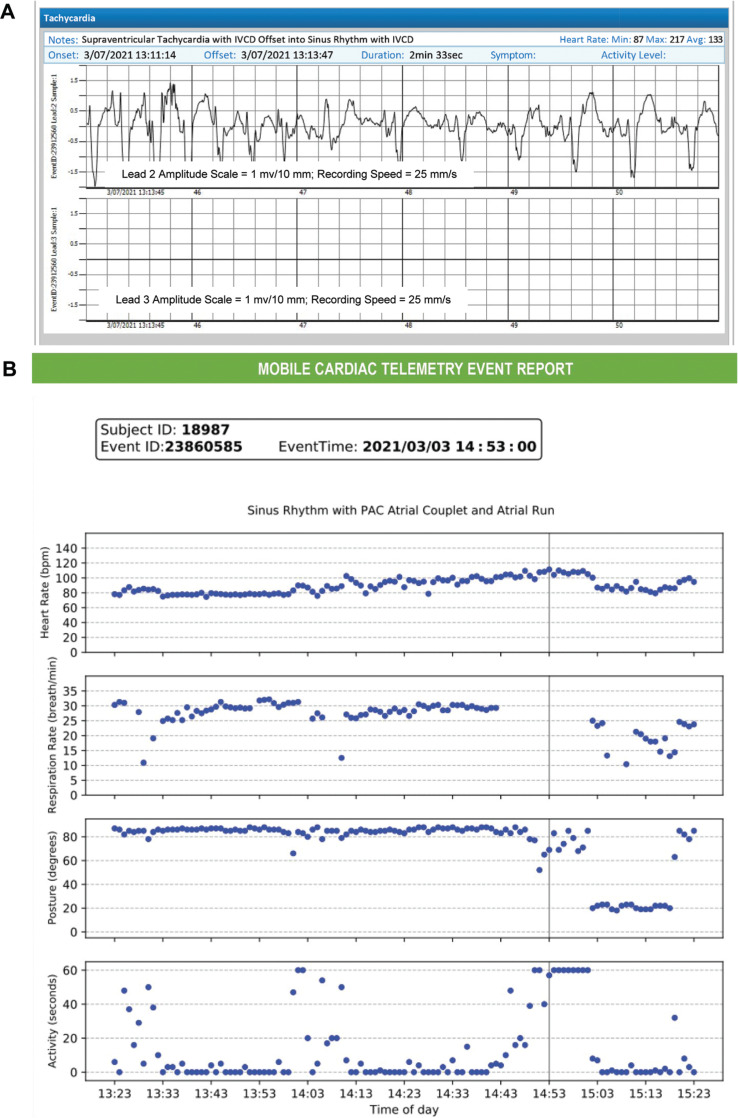
**A:** Sample event report consisting of a 6-s electrocardiogram strip with rhythm interpretation. **B:** Biometrics data include heart rate, respiratory rate, posture, and activity trends. The biometrics trend information is provided in 1-min intervals (X-axis in panel **B**) 90 min before and 30 min after the event (gray vertical line).

Event reports are generated for subject-initiated recordings or when arrhythmias are detected. They contain ECG data, the technician’s interpretation regarding the arrhythmia, and/or notes around the subject-reported symptoms **(Figure 2A)**. The event report also contains biometric data around the timeframe when the event occurred (see **Figure 2**). The HR, RR, posture, and activity (including sleep and inactive states) trend information provide objective contextual details about the event. Active, sleep, and inactive states are based on a combination of thresholds from the activity and posture measurements. There are no paper diaries.

Similar to the event reports, a daily report provides a summary of the subject’s arrhythmic and subject-reported events for the day, along with the biometric trends. At the end of device wear for each subject, an EOU report is provided that contains an assessment of the subject’s wellness in addition to the rhythm and biometric information summary (see **Figure 3**). These trended data provide insights to the physician regarding the longitudinal health status of the patient during the monitoring period.

The study was a multicenter, prospective study (NCT04754204) that recruited patients from cardiology practices in the United States. The centers enrolled consenting subjects in a sequential manner to avoid selection bias. For all arrhythmic events or subject-reported symptom events, for a maximum monitoring period of 30 days, investigators received an event report. Investigators initially reviewed the ECG data alone and answered a questionnaire on whether they found the ECG rhythm or symptom information useful. The investigator then reviewed the associated biometrics data to answer a second series of questions to document whether the biometric data were useful in clarifying or changing their ECG- or symptom-based treatment plan. These modifications included changes to medication, follow-up plan, and lifestyle. At the end of device use, investigators received an EOU report and answered additional questionnaires to understand how they used the longitudinal wellness information.

Patients aged ≥21 years with an indication for MCT monitoring were enrolled. Patients were excluded if they had an implantable cardiac device, wearable cardioverter defibrillator, Holter monitor, wearable event recorder, or another MCT device at the same time. Other exclusion criteria were a skin condition preventing the patient from wearing the AMS device, being hospitalized at the time of screening, being non-ambulatory, pregnancy, and participating in another study. The protocol was approved by a central or local institutional review board, and all subjects provided informed consent.

The following endpoints were evaluated:

- The association of biometrics data with arrhythmias and subject-reported symptoms in order to determine whether significant relationships existed among biometrics data, arrhythmias, and symptoms
- The demonstrated clinical utility provided by adding biometric information to the event reports containing ECG-only recordings and subject-reported symptoms in order to determine whether physicians were able to interpret the biometrics data and to assess the usefulness of this data
- The overall usefulness of the longitudinal biometrics data from the EOU report for better patient management decisions, including specifically to determine whether these wellness data provided additional benefits beyond the arrhythmia and symptom data

Event-, subject-, and physician-level analyses were performed. Demographic, clinical, follow-up, and biometric data were presented as mean ± standard deviation values and, for skewed distributions, as median and interquartile range (IQR) values. Arrhythmias analyzed included ectopic atrial and ventricular beats, AF, supraventricular tachycardia (SVT), intraventricular conduction disorders (IVCDs), ventricular tachycardia (VT), pause, and second-/third-degree atrioventricular block (AVB). Odds ratios (ORs) were used to quantify the strength of the associations among the arrhythmias of interest, subject-reported symptoms, and the activity biometrics. A *t* test with Holm–Bonferroni correction was used to compare HR and RR values between the various arrhythmias of interest and subject-reported symptoms. Physician responses to the event report and EOU report questionnaires were reported at event, subject, and physician levels. If the addition of the biometric data caused modifications to a subject’s treatment plan, these changes were analyzed in the context of each of the biometrics (activity, body position, sleep, and RR). All analyses were conducted using RStudio 1.2.1335 (RStudio Inc., Boston, MA, USA). *P* < .05 was considered statistically significant.

## Results

From December 2020 through July 2021, 583 patients were enrolled by 27 physicians from 18 U.S. cardiology practices. **Table 1** includes the reasons for monitoring and basic patient demographics. Based on the MCT prescription, the median prescribed length was 30 days (IQR, 14–30 days). To generate the wellness report, ≥24 h was required. Of the 583 subjects, 537 (92%) wore the AMS for >24 h. The median number of days these subjects used the device was 21 days (IQR, 12–30 days). Of the 537 subjects who wore the device for >24 h, EOU reports were published for 535 subjects. Insufficient trend information prevented the creation of EOU reports for the remaining 2 subjects.

A total of 5,831 event reports were generated for 535 subjects. Arrhythmias of interest were detected in 365 (68%) subjects. Ectopic beats, AF, SVT, IVCDs, VT, pause, and second-/third-degree AVB were detected in 53%, 15%, 13%, 11%, 3%, 2.6%, and 1.9% of the subjects, respectively. Overall, 313 (59%) subjects reported ≥1 symptom. The commonly reported symptoms were heart racing (25%), light-headedness (22%), shortness of breath (21%), fatigue (19%), a skipped heartbeat (18%), and chest discomfort (14%).

### Activity and arrhythmias

**Figure 4:**
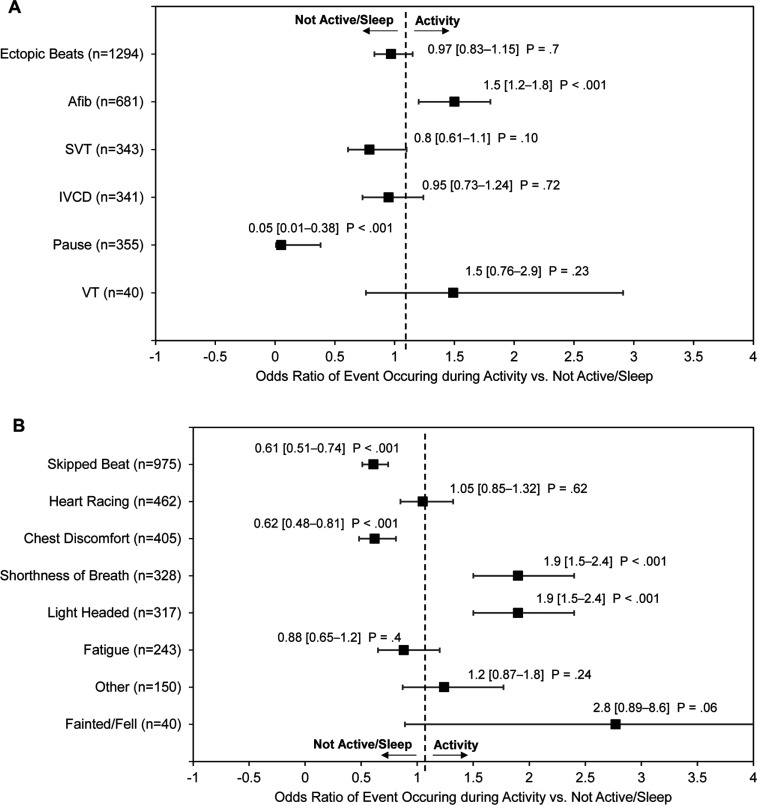
Forrest plot of **(A)** arrhythmias and **(B)** subject-reported symptoms occurring during activity versus inactive/sleep. Shown are the point estimate of the odds ratio (OR) and the 95% confidence interval along with the *P* values. An OR of 1 indicates that a particular arrhythmia or symptom has an equal likelihood of occurring when the subject is active or inactive/sleep in comparison to other arrhythmias or symptoms. Note, in **A**, the OR value for the second-/third-degree atrioventricular block (AVB) is not displayed since none of the AVB events occurred during activity.

When associating the activity biometrics with arrhythmias for the group as a whole, 57% of the arrhythmias were detected when subjects were inactive, 25% were detected when subjects were active, and 18% occurred while sleeping. AF was 1.5 times more likely to occur during activity compared to when the subject was inactive/sleeping (OR: 1.5; 95% confidence interval [CI], 1.2–1.8; *P* < .001; **Figure 4A**). In contrast, second-/third-degree AVB always occurred when the subject was inactive/sleeping (*P* < .001). Similarly, pauses were 19 times more likely to occur when the subject was inactive/sleeping (OR, 19; 95% CI, 2.6–137; *P* < .001; **Figure 4A**). Ectopic beats and IVCDs had a point estimate OR of approximately 1, indicating that these events had an equal likelihood of occurring when the subject is active or not active/sleep. Interestingly, SVT had a point estimate OR of 0.8, indicating that these events were likely to occur when the subject was inactive/sleep, although this likelihood was not statistically significant (OR, 0.8; 95% CI, 0.73–1.24; *P* = .10). In contrast, VT showed a propensity for occurring when subjects were active, although this likelihood was also not statistically significant (OR, 1.5; 95% CI, 0.76–2.9; *P* = .23).

### Heart rate and arrhythmias

When associating onset HRs with arrhythmias, the median HR of 58 bpm (IQR, 62–82 bpm) at the onset of pause events was significantly lower (*P* < .001) than the onset HR of other arrhythmias. In contrast, the median onset HR at the onset of SVT (98 bpm; IQR, 78–124 bpm) and AF (96 bpm; IQR, 78–121 bpm) events was significantly higher (*P* < .001) than the onset HR of other arrhythmias.

### Respiratory rate and arrhythmias

Interestingly, the median RR of 19 breaths/min (IQR, 17–21 breaths/min) at the onset of SVT was significantly higher (*P* < .001) than the RR of other arrhythmias. In contrast, the median RR of 16 breaths/min (IQR, 13–16 breaths/min) at the onset of a pause event was significantly lower (*P* < .001) than that of other arrhythmias.

### Activity and symptoms

When associating activity with symptoms, 63% of symptoms were reported to have occurred when subjects were not active, 27% occurred when they were active, and 10% occurred when they were sleeping. Skipped heartbeats and chest discomfort were 1.6 times more likely to occur when the subject was inactive/sleeping compared to being active (OR, 1.6; 95% CI, 1.3–2.0; *P* < .001; **Figure 4B**). In contrast, shortness of breath and light-headedness were nearly 2 times more likely to occur when the subject was active (OR, 1.9; 95% CI, 1.5–2.4; *P* < .001; **Figure 4B**). Although fainting/fall symptoms were approximately 3 times more likely to occur when subjects were active, this did not reach statistical significance (OR, 2.8; 95% CI, 0.89–8.6; *P* = .06). The remaining reported symptoms showed no significant relationship with activity.

### Heart rate and symptoms

When evaluating HR relation with symptoms, the median HR of 75 bpm (IQR, 66–85 bpm) for skipped beats was significantly lower (*P* < .001) than those for other symptoms. In contrast, the median HR for the heart-racing symptom (91 bpm; IQR, 79–108 bpm) was significantly higher (*P* < .001).

### Respiratory rate and symptoms

The median RR of 21 breaths/min (IQR, 15–24 breaths/min) for shortness-of-breath symptoms was significantly higher (*P* < .001) than the median RR for other symptoms.

### Physician-reported utility of the biometric information to ECG-only and symptom-only assessments

A total of 27 physicians completed 2713 event report questionnaires from 520 subjects. When presented with just the initial ECG and associated symptoms, physicians labeled 17% of the events as occurring while subjects were active, 5% of the events as occurring while subjects were sleeping, and 3% as occurring while subjects were tachypneic. In contrast, when physicians reviewed the ECG along with the biometrics data, they were able to ascertain that 70% (*P* < .001) of the events occurred while the subjects were active, 55% (*P* < .001) occurred during sleeping, and 39% (*P* < .001) occurred while subjects were tachypneic.

**Figure 5:**
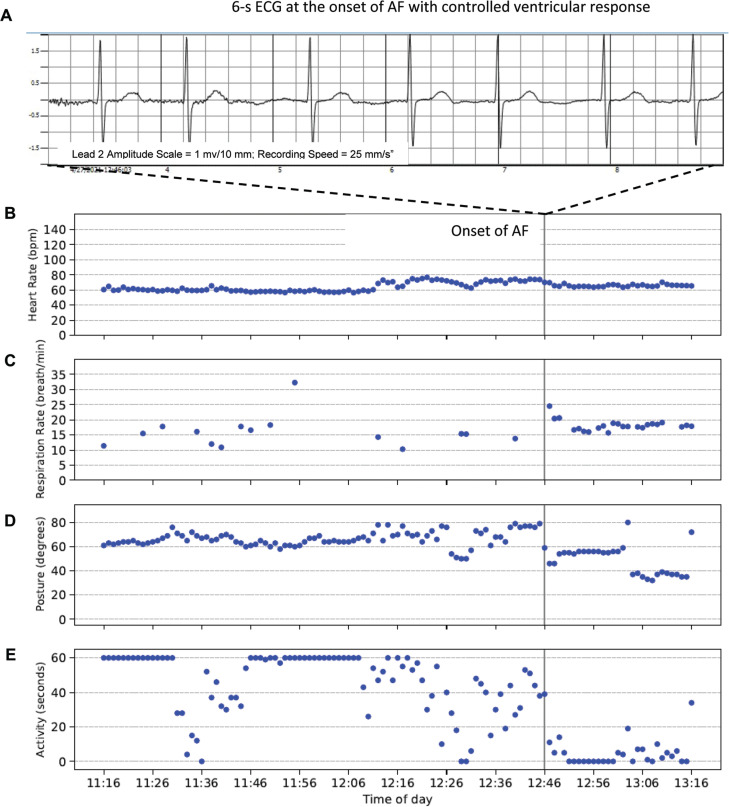
**A:** A 6-s electrocardiogram tracing at the onset of atrial fibrillation (AF) with controlled ventricular response. **B–E:** The biometric trend data. The gray vertical line depicts the time stamp of the onset of the AF event. Heart rate **(B)**, respiratory rate **(C)**, posture **(D)**, and activity **(E)** trend data are shown in 1-min intervals 90 min before and 30 min after the AF onset (gray vertical line). Note, active is defined as posture > 35° and activity of ≥30 s/min.

The value of these biometric data is illustrated in **Figure 5** with a case of AF onset with a controlled ventricular response. Minutes before the onset of AF, the subject showed a decreasing trend in activity **(Figure 5E)**. At the onset of the AF, the subject was inactive (sitting; **Figure 5E**) and tachypneic (RR, 25 breaths/min; **Figure 5C**). The subject returned to being active after approximately 30 min of being inactive. Based on these biometric trend data, the physician initiated changes in the medication, follow-up plan, and subject’s lifestyle.

Overall, when ECG data were augmented with biometric data, 96% of the physicians altered the patient’s treatment plan compared to standard MCT ECG data for ≥1 patient. These changes included adjustments to medications, follow-up schedule, and/or the subject’s lifestyle. For example, the combination of sudden changes in HR and activity biometrics was used to make changes to β-blocker dosages or to provide reassurance if symptoms were reported. RR and sleep data were used in the setting of tachy-/bradyarrhythmia management of medication titration and to separately assess the need for sleep apnea testing. In combination, treatment plans were changed for a total of 64% of the subjects, which included changes to medications, the follow-up plan, and patient lifestyle in 16%, 17%, and 64% of the subjects, respectively.

When determining which biometric data physicians found useful in the recommended lifestyle changes, information on activity was considered most useful in 62% of the subjects, followed by information on body position, sleep, and RR in 46%, 36%, and 30% of the subjects, respectively. The change recommendations included increased activity/exercise, explanation of relationship of events to activity and body position, changing the sleep position, changing the sleep pattern, stress reduction, reduction of caffeine intake, and subject reassurance.

The event-level analysis of physicians’ use of the biometrics data plans is shown in **Table 2**. For example, for VT events, the treatment plan was modified compared to ECG alone in 90% of the events, with activity and body position being the most useful. In contrast, RR was a factor in treatment modification in only 21% of the events. Similarly, when the subject reported fainting or a fall, physicians used biometrics data to adjust treatment in 88% of the events compared to ECG alone. In these patients, the activity data were the most helpful (86%) and the sleep data were the least useful (29%).

Longitudinal wellness information for the duration of AMS use was provided for 535 subjects in the form of an EOU report. The median nocturnal HR for the entire cohort was 65 bpm (IQR, 59–73 bpm) and was significantly lower (*P* < .001) than the median daytime HR of 74 bpm (IQR, 67–83 bpm). Interestingly, the median daytime and nocturnal HR across the monitoring period were stable (*P* = 1). Similar trends were seen for median daytime and nocturnal RR. The overall median RRs during the day and during the night were 17 breaths/min (IQR, 16–19 breaths/min) and 15 breaths/min (IQR, 14–18 breaths/min), respectively (*P* < .001).

Overall, the group was active for a median time of 3.3 h/day (IQR, 1.6–5.4 h/day). Over the 30-day wear period, the daily activity duration varied by a mean of <15 min (12 ± 17 min). When analyzing the overall sleep duration, the median was 6.4 h/day (IQR, 3.9–8.6 h/day), and this duration varied by a mean of <15 min (14 ± 10 min).

A total of 27 physicians completed EOU report questionnaires for 534 subjects. Ninety-six percent of the physicians found the EOU wellness information useful in at ≥1 patient to better understand the circumstances associated with the arrhythmia diagnosis or subject-reported symptoms (52% of the total subjects).

After being provided with wellness information, 67% of the physicians modified the treatment plan for ≥1 patient. These changes were made for 24% of the total subjects, which included changes to medications, the follow-up plan, and lifestyle in 11%, 11%, and 22% of subjects, respectively. For the 91 subjects who had no reported arrhythmias or symptoms, physicians reported the wellness report was helpful for better understanding the patient status in 38 subjects (42%).

**Figure 6:**
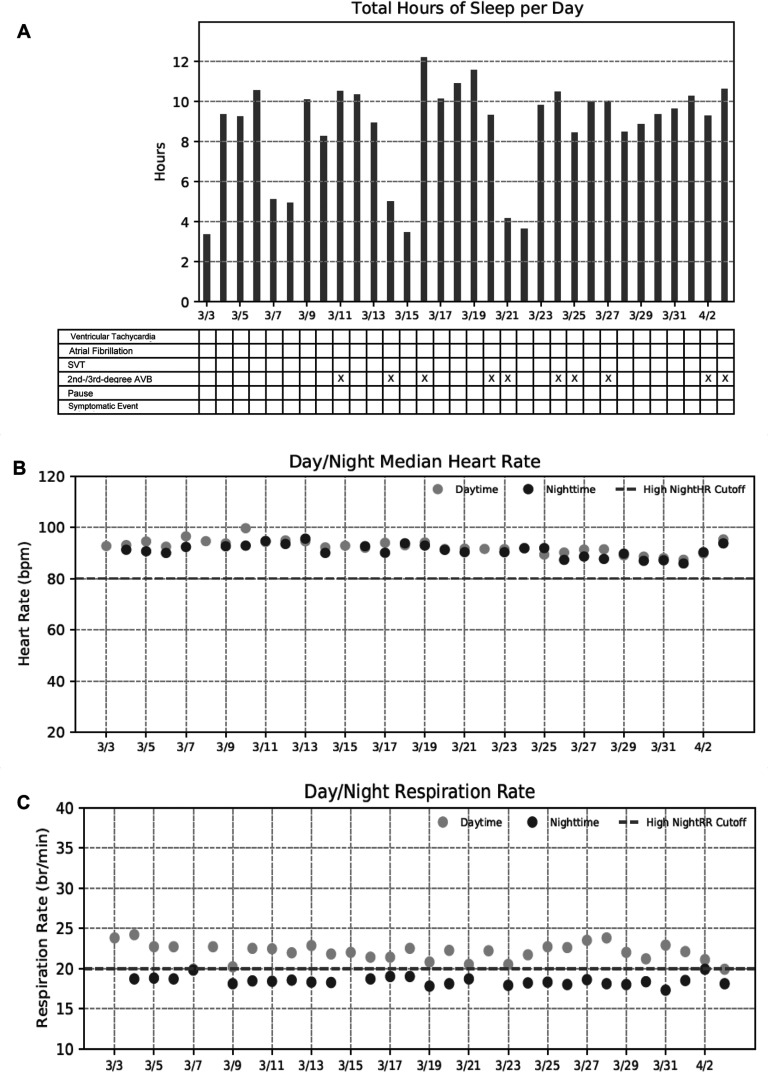
Select wellness trend data from the end-of-use report for 1 subject. **A:** Sleep duration for each day of monitoring along with a summary of arrhythmias and symptomatic events. Over several days during the monitoring period, the subject experienced several second-/third-degree atrioventricular block when sleeping as detected by the arrhythmia monitoring system device. Note, sleep is defined as posture ≤ 35° and activity of <12 s/min. **B, C:** Median heart rate and respiratory rate data are reported for each monitoring time day and night.

Select wellness information from the EOU report was used to exemplify the value of these data **(Figure 6)**. The example subject experienced several episodes of second-/third-degree AVB while sleeping **(Figure 6A)**. The subject also experienced a consistently elevated median HR of approximately 90 bpm during each night **(Figure 6B)**. This subject also had an intermittent elevated RR of ≥20 breaths/min during the day and an increased RR of >18 breaths/min during the night **(Figure 6C)**. The physician shared these longitudinal data with the patient and reported “addressing sleep apnea as the potential cause” was the next step.

Of the 541 subjects who wore the AMS, a total of 28 subjects (5.4%) discontinued use due to skin irritation. The median wear time to discontinuation from skin irritation was 14 days (IQR, 9–21 days). Twenty-eight other patients (5.4%) reported some level of skin irritation that did not lead to discontinuation.

## Discussion

This prospective, multicenter study evaluated the clinical utility of combining physiologic parameters (biometrics) with ECG and patient-reported symptom data in the largest prospective MCT study published to date. This is the first large trial to evaluate the relationships between device-recorded biometrics and arrhythmia event and symptoms.

Comparing decisions from ECG + biometrics data (both from event and EOU reports) to ECG alone, we found that 96% of physicians made changes to ≥1 patient’s treatment plan. The biometrics-based treatment plan changes involved 64% of all patients (n = 535) and included modifications to medications, follow-up plans, and lifestyle in 18%, 19%, and 63% of the subjects, respectively. Furthermore, for the subgroup of subjects with a detected arrhythmia or reported symptom (n = 444), physicians reported that the biometrics data helped them to better understand the circumstances associated with the event in 69% of these subjects.

This is the first study to report the associations of arrhythmias and symptoms with biometrics data in patients who require MCT monitoring. We found that, among the various arrhythmias and symptoms, AF, shortness of breath, and light-headedness had a significant likelihood of occurring during activity. In contrast, second/third-AVB, pauses, skipped beats, and heart-racing events were more likely to occur when subjects were inactive/asleep. Providing objective physiologic data from the AMS monitor reduces or eliminates the reliance on the subjective patient diaries and provides more in-depth contextual information around provocative factors and patient responses, enhancing the interpretation of both arrhythmic events and symptoms.

New or increasing symptoms during therapy titration may prompt an investigation with ambulatory cardiac monitoring. In such cases, the AMS HR and biometrics trend data can aid the physician in better therapy titration. The current study found that the onset of AF had a median heart rate of 96 bpm, which was significantly higher than the HR at the onset of other arrhythmias and 1.5 times more likely to happen during activity. Based on this, physicians used the activity assessment at a higher frequency (91% of the events; **Table 2**) than other biometrics parameters when modifying the treatment plan for patients with AF.

While most cardiac monitors are prescribed solely for diagnostic purposes, often, no arrhythmias are detected or there is no clear relationship between symptoms and arrhythmias. The ability to review daily biometrics, align arrhythmias and symptoms with patient circumstances, and the availability of trending data allow for added clarity in outpatient monitoring. The wellness longitudinal report at the end of use provides information not available with current ECG-only–based MCT. For example, the wellness report contains daytime and nocturnal HR trending data over the entire wear period. This information was found to be useful in assessing the response to medication titration and can be a tool to determine medication compliance.

Ambulatory daily RR trends and elevated sleeping RR have been identified as important risk markers for heart failure and sleep apnea.^4,5^ However, no previous study has reported the relationships between arrhythmic or symptomatic events and RR. In this study, the RR at the onset of SVT and shortness-of-breath events were significantly higher than the RR of other arrhythmias and other reported symptoms. In contrast, the RR at the onset of pause events was significantly lower than that seen at the onset of other arrhythmias. Interestingly, physicians modified the treatment for 76% **(Table 2)** of pause events when they had RR data available. These results exemplify the fidelity of the AMS biometrics data to capture the interactions of the cardiopulmonary system, which can then allow physicians to modify their treatment plan accordingly.

One of the objectives of this study was to determine if the biometric data alone provided any value. In the current study, 91 (17%) of the 535 subjects did not experience an arrhythmia or report any symptoms. Typically, for these subjects, MCT monitoring would be considered non-diagnostic. In this analysis, however, physicians were able to use the longitudinal wellness information to better understand the patient status. Physicians reported that trend data were important in 38 (42%) of these 91 subjects. Even in subjects with no arrhythmic or symptomatic events, these longitudinal wellness data add value by providing insights into the patient’s overall wellness and health status by reporting total daily activity time and the longitudinal pattern of a person’s sleep time rather than just data from the 1–3 nights typical of a sleep study or self-reported data.

Lastly, patients are always looking for an explanation for their symptoms or arrhythmias. Being able to discuss the circumstances around their events using the biometric information allows for a more robust discussion. In several cases, these longitudinal biometric data were harbingers of other cardiovascular risk and comorbidities. For example, as seen in **Figure 6B**, an elevated nocturnal HR, where HR decreased by <10% from the daytime HR, has been shown in previous studies as an important predictor of cardiovascular and non-cardiovascular risk.^6,7^

### Limitations

Several limitations in this investigation should be reviewed. Sleep was defined using a combination of thresholds of the activity and posture biometrics. Such a binary definition (asleep/not asleep) may not accurately capture the actual sleeping state of the subject. Additionally, individual patient compliance to medications and/or medication changes and dosages, follow-up plan, and exact lifestyle changes were not captured during the study. The purpose of this study was to document the relationship between the initial arrhythmias and symptoms and biometric data. Further comparisons, which were out of the scope of this non-randomized study, would be required to document the impact of any physician-prescribed changes and the associated patient outcomes. It bears repeating that this investigation was designed to determine whether biometric information added to a traditional MCT device provides value to the prescriber, as this has not previously been studied.

The high level of physician engagement with the biometric data seen herein may have been driven by their participation in a clinical study that included physicians who were already high prescribers of MCT. Any bias, whether conscious or unconscious, introduced by participation in a study is unavoidable and may have led to overvaluing the benefit of biometric information. The level of data engagement might be reduced in a non-study setting or among physicians who place less value on MCT. However, compared to the sporadic information from diaries that is usually available, objective biometric data should be useful.

The AMS device does not record HR variability, corrected QT data, or ST-segment/T-wave changes.

## Conclusions

This large prospective, multicenter study was the first to report the associations of arrhythmias and symptoms to biometrics data in patients who require MCT. Importantly, all physicians were able to interpret the biometrics data and apply the biometric information to improve patient management decisions. These decisions included assistance with the arrhythmia diagnosis or subject-reported symptoms and use of this information to improve patient management decisions. Next-generation MCT, by providing multiple biometric parameters along with ECG data, improves physicians’ ability to make patient management decisions. This added functionality and clarity may replace simple ECG-based monitoring.

## Figures and Tables

**Figure 3:**
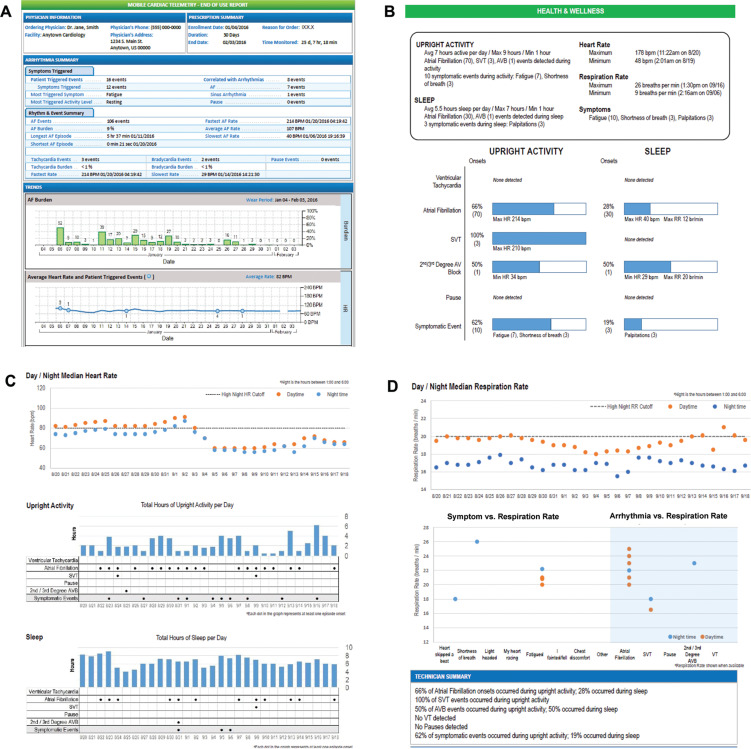
End-of-use report consisting of the arrhythmia and symptom summary, along with the **(A)** atrial fibrillation burden and heart rate (HR) trends and **(B–D)** wellness information. **B** contains the summary statistics for upright activity duration, sleep duration, HR, and respiratory rate (RR). Also displayed is the summary of the arrhythmia and symptom onsets experienced by the subject during the monitoring period. **C** contains the median HR trend data during the day (daytime) and during the night (nocturnal) as well as the duration of upright activity and sleep for each of the monitored days along with any arrhythmia or subject-reported symptom onsets. **D** contains the median daytime and nocturnal RR trends and graphical representation of the median RRs during subject-reported symptom onsets and arrhythmia onsets, and the technician summary section provides a concise review of the percentage of the time arrhythmias or symptoms occurred during activity and sleep.

**Figure 3:**
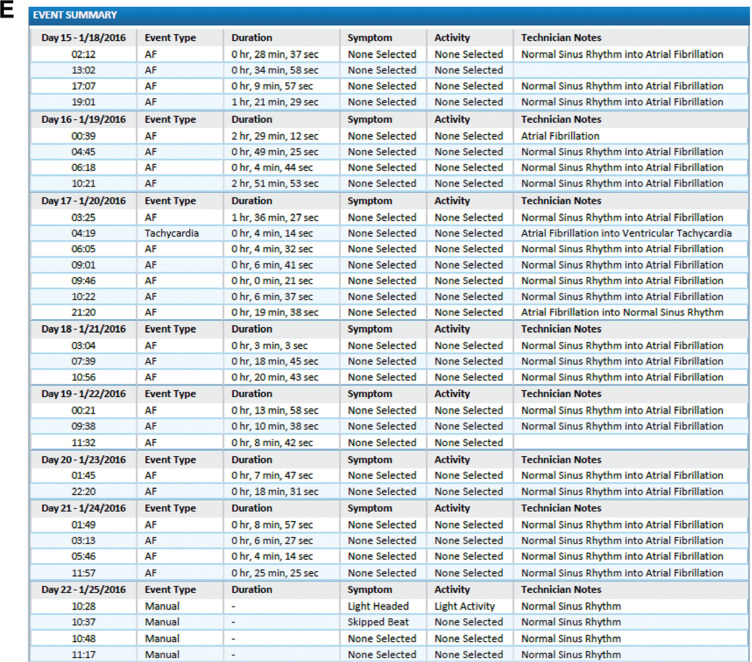
End-of-use report consisting of the arrhythmia and symptom summary, with **(E)** an event summary of the arrhythmia and/or symptoms detected during the monitoring period along with a chronological listing of the events for the entire monitoring period.

**Table 1:** Patient Baseline Characteristics at Study Enrollment

N = 583	
Age, years	63 ± 16
Male, n	249 (43%)
MCT monitoring reason	
Palpitations, n	230 (39%)
AF, n	121 (21%)
Syncope/Pre-syncope, n	86 (15%)
SVT/tachycardia/bradycardia, n	62 (12%)
Other, n	84 (14%)
Body mass index, kg/^2^	31 ± 8
Height, in	67 ± 7
Weight, lbs	195 ± 52
Ejection fraction, % (n = 284)	58 ± 9
History, n	
Palpitations	406 (70%)
Hypertension	375 (64%)
Hyperlipidemia	319 (55%)
Dizziness	240 (41%)
Smoking	221 (38%)
Syncope	101 (17%)
SVT	88 (15%)
Bradycardia	87 (15%)
Sleep apnea	73 (13%)
Heart failure	66 (11%)
Coronary artery bypass graft	64 (11%)
Prior ablation	63 (11%)
Stroke	61 (10%)
Myocardial infarction	40 (7%)
Chronic kidney disease	34 (6%)

*Abbreviations:* AF, atrial fibrillation; MCT, mobile cardiac telemetry; SVT, supraventricular tachycardia.

**Table 2:** Analysis of the Use of Biometric Data to Modify the Treatment Plans Based on Arrhythmias and Symptoms Reported

Treatment Plan Change and Biometrics (n = 2713 events)						
	Total Events, n	Treatment Plan Changed, n (%)	Treatment Plan Change Using Activity (%)	Treatment Plan Change Using Body Position (%)	Treatment Plan Change Using Sleep (%)	Treatment Plan Change Using Respiration Rate (%)
Arrhythmia						
Ectopic beat	590	304 (51)	98	74	56	43
AF	341	224 (66)	91	67	71	33
SVT	174	114 (66)	99	73	51	43
IVCD	117	65 (56)	97	83	69	66
Second-/third-degree AVB	35	22 (63)	95	95	86	9
Pause	34	22 (62)	91	86	82	73
Ventricular tachycardia	21	19 (90)	89	84	42	21
Symptom						
Skipped beat	385	238 (62)	97	66	46	41
Heart racing	284	170 (60)	99	72	62	45
Shortness of breath	217	99 (46)	97	79	65	49
Light headed	214	118 (55)	96	78	51	44
Chest discomfort	199	111 (55)	97	81	77	61
Fatigue	154	88 (58)	99	74	57	55
Other	106	58 (55)	98	74	64	67
Fainted/fell	8	7 (88)	86	57	29	57

*Abbreviations:* AF, atrial fibrillation; AVB, atrioventricular block; IVCD, intraventricular conduction disorders; RR, respiratory rate; SVT, supraventricular tachycardia. The “total events” column provides the number of specific arrhythmias and symptoms evaluated by physicians. The “treatment plan changed” column provides the number and percentage of events for which the treatment plan (medication, follow-up plan, and/or subject lifestyle) was changed. The remaining columns provide the percentage of events for which treatment plan was changed using the activity, body position, sleep, and RR biometrics.
